# Effects of a period without mandatory physical training on maximum oxygen uptake and anthropometric parameters in naval cadets

**DOI:** 10.1371/journal.pone.0251516

**Published:** 2021-06-02

**Authors:** Álvaro Huerta Ojeda, Guillermo Barahona-Fuentes, Sergio Galdames Maliqueo

**Affiliations:** Grupo de Investigación en Salud, Actividad Física y Deporte ISAFYD, Escuela de Educación Física, Universidad de Las Américas, sede Viña del Mar, Chile; West Virginia University, UNITED STATES

## Abstract

The effects of a period without physical training on the civilian population are well established. However, no studies show the effects of a period without mandatory physical training on maximum oxygen uptake (VO_2_ max) and anthropometric parameters in naval cadets. This study aimed to investigate changes in VO_2_ max and anthropometric parameters after 12 weeks without mandatory physical training in naval cadets. The sample was 38 healthy and physically active naval cadets. The measured variables, including VO_2_ max and anthropometric parameters, were evaluated through the 12-minute race test (12MRT) and the somatotype. Both variables had a separation of 12 weeks without mandatory physical training. A t-test for related samples was used to evidence changes between the test and post-test; effect size was calculated through Cohen’s d-test. Distance in 12MRT and VO_2_ max showed significant decreases at the end of 12 weeks without mandatory physical training (p < 0.001). Likewise, the tricipital skinfold thickness and the endomorphic component showed significant increases (p < 0.05). 12 weeks without mandatory physical training significantly reduces the VO_2_ max in naval cadets. Simultaneously, the same period without physical training increases both the tricipital skinfold thickness and the endomorphic component in this population.

## Introduction

Increased physical capabilities through strength training [1, 2] and aerobic capacity [3] have been associated with health, quality of life, and sports performance benefits [1–3]. In this sense, people included in strength training have shown neuronal and morphological adaptations [4]; these two adaptations, generated by strength training, allow for the improvement of both the metabolic health [5] and the quality of life of people [6]. At the same time, aerobic training has reported significant decreases in cardiovascular risk factors [7], as well as an increase in maximum oxygen uptake (VO_2_ max) [3]. Specifically, the VO_2_ max has a direct association with the quality of life of people [8]. These morphological and metabolic changes, triggered by force training or aerobic training, are experienced by both the civilian population [9] and the military and naval population [10–12]; in the latter, they provide specific physical characteristics that allow missions to be carried out efficiently and with a low risk of injury [13].

Scientific evidence shows that physical training acts as a physiological stressor, increasing energy expenditure [14], anabolic hormone concentrations [15], arterial diameter, and blood flow [16]. These responses to physical training contribute to a physiological adaptation of the body [17], specifical adaptations of muscles [18], and bone tissues [19]. In this sense, a recently published meta-analysis showed the benefits of eccentric strength training through isoinertial devices; the study results showed increases in strength, power, and muscle size with this training [20]. Concerning aerobic training, these stimuli have been considered as the primary method to improve markers of cardiorespiratory fitness, mainly VO_2_ max [21]. Additionally, physical training carried out regularly, and with the principles of intensity, volume, and frequency, will minimize muscular fatigue [22] and favor the physiological adaptations of the body [17]. Despite the above, there is also a transition phase in sports periodization [23]; this stage corresponds to the interruption of physical training [24], which can be short term (less than four weeks) or long term (more than four weeks) [25]. However, if professionals do not control the transition phase, there is a high probability of provoking a detraining [25]. In this way, a period without physical training can generate a partial or total loss of morphological adaptations, physiological adaptations, and physical performance [26], as well as cause alterations in the psychological well-being of the population [27].

The sports transition phase is an opportunity for the physical recovery of athletes [23]. However, there are unplanned situations that generate periods of non-physical training in the population [28–30], for example, the period of vacation experienced by students each year [28] or the current period of confinement generated by COVID-19 [30]. Regardless of the reasons, an extended time-period without physical training has been shown to negatively influence athletes’ body composition [23], increasing fat mass and decreasing lean mass [31–33]. It has also been shown that a period without physical training of fewer than eight weeks leads to a decrease in muscle cross-section [34], decreases in maximum strength [35], and a reduction in VO_2_ max in both the civilian [36] and naval [37] populations.

Currently, naval personnel has been the subject of several research studies [38, 39]. One of the reasons for the growing number of investigations in this sample is that the Chilean Navy comprises more than 25,000 personnel. Of this number, 9.6% (equivalent to 2,400 personnel) corresponds to naval officers, all trained at the Arturo Prat Naval Academy [40]. These figures show several aspects, such as the high number of officers [40] and, therefore, the need for this population to be studied from a psychological [11, 13], health [12] and physical [10, 38] performance perspective. This last dimension includes the transition phase considering that we hypothesize that naval cadets decrease their physical condition, associated with VO_2_ max and anthropometric parameters, after a period without mandatory physical training; thus, with correctly applied training loads, physical fitness loss in this phase could be avoided [23–25].

Despite the existence of studies showing a decrease in the physical condition and anthropometric parameters after a period without physical training in some segments of the population [23, 31–33], the available evidence in the naval population is scarce and limited [37]. Likewise, and as far as knowledge goes, no studies evidence the effects of periods without physical training on VO_2_ max and anthropometric parameters in naval cadets from 18 to 25 years old. Consequently, this study aimed to evidence the changes in VO_2_ max and anthropometric parameters after 12 weeks without mandatory physical training in naval cadets from 18 to 25 years old.

## Materials and methods

### Research design

**Fig 1:**
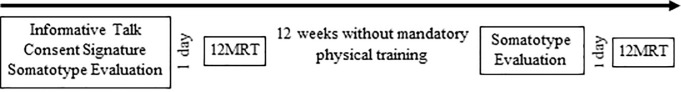
Research design. 12MRT: 12-minute race test.

This study was empirical research with a manipulative, quasi-experimental strategy with a longitudinal design with repeated means [41]. To highlight the changes in VO_2_ max and anthropometric parameters, the 12-minute race test (12MRT) and the somatotype were evaluated 12 weeks apart, a period without mandatory physical training (Fig 1).

### Procedures

As a first action, all participants who voluntarily accepted to be part of the study (non-probabilistic sample) were recruited. The purpose and procedures of the study were indicated in an informative talk. The inclusion criteria were that all participants should be healthy, physically active [21] and between 18 and 25 years of age, while the exclusion criteria were: prevalence of musculoskeletal injuries, pre-existing cardiac pathologies, abnormal respiratory and cardiac responses during the familiarization period and inability to perform the 12MRT. All participants were asked not to engage in physical training that would generate nervous or musculoskeletal fatigue 48 hours before the measurements and refrain from ingesting caffeine or any substance that could increase their metabolism during the assessment. Finally, only those participants who signed informed consent were subjected to 12MRT and somatotype evaluations.

### Participants

Thirty-eight healthy and physically active naval cadets volunteered to participate in this study (Table 1). The type of sampling was non-probabilistic for convenience. All participants were informed of the study objective and possible risks of the experiment. Indeed, all participants signed the informed consent form before the implementation of the protocols. The informed consent and the study were approved by the Human Research Committee of the University of Las Americas (registry number CEC-FP-2020011). The informed consent and the study were conducted under the Declaration of Helsinki (WMA 2000, Bošnjak 2001, Tyebkhan 2003), which sets out the fundamental ethical principles for research with human subjects.

**Table 1:** Characterization of the participants.

	Women (n = 8)	Men (n = 30)	All (n = 38)
	mean ± SD (min–max)	Mean ± SD (min–max)	mean ± SD (min–max)
**Age (years)**	21.0 ± 1.51 (19–23)	20.5 ± 1.22 (18–24)	20.6 ± 1.28 (18–24)
**BMI (kg/m**^**2**^**)**	21.9 ± 1.79 (20.2–25.5)	22.7 ± 1.69 (20.4–26.7)	22.5 ± 1.72 (20.2–26.7)
**% Fat**	23.3 ± 4.7 (18.5–33.1)	12.6 ± 2.2 (9.3–18.1)	14.9 ± 5.2 (9.3–33.1)
**VO**_**2**_ **max (mLO**_**2**_**·kg**^**–1**^**·min**^**–1**^**)**	46.7 ± 3.9 (42.6–51.5)	59.3 ± 4.7 (50.9–65.5)	56.6 ± 6.9 (42.6–65.5)

SD: standard deviation; kg/m^2^: kilograms per square meters; min: minimum; max: maximum; %: percentage; VO_2_ max: maximum oxygen uptake; mLO_2_·kg^–1^·min^–1^: milliliters of oxygen per kilogram of body mass per minute.

### Somatotype evaluation

The somatotype corresponds to the shape of the human body. It is obtained by analyzing the arm and leg’s circumferences, the humerus and femur’s diameters, four skinfolds (tricipital, subscapular, supra-iliac, and mid-calf), and the weight and height of a person. Body shape can be represented two-dimensionally through the somatochart or three-dimensionally through the compogram; the latter representation corresponds to three numerical values representing the endomorphic, mesomorphic, and ectomorphic components of a participant (always in that order) [42]. To represent a participant’s morphology, Berral [42] recommends using both the somatochart and the compogram since using only the somatochart can generate an error in interpreting the results; for example, values 3–5–3 and 4–6–4 would be represented with the same point on the somatochart [42].

#### Body mass and height

The method used to determine the participants’ somatotype was proposed by Carter & Heath [43]. The body mass (kg) was evaluated through a Tanita Inner Scan BC-554® digital scale, with the participants barefoot, in shorts, and wearing a light shirt. The height was measured through a Seca® stadiometer from the feet to the vertex (Frankfort plane) [44].

#### Circumferences

Arm and leg circumferences, humeral and femoral diameters, and skin folds were evaluated with the FAGA SLR® anthropometric kit. The circumference of the right leg was evaluated in this segment’s bulkiest area, in a standing position and with the gastrocnemius relaxed; in contrast, the circumference of the right arm was evaluated in the bulkiest area of the contracted biceps; this evaluation was performed standing with the elbow in front and bent at 90 [43].

#### Diameters

The humeral epicondyle distance was considered the humerus’s diameter, which is the distance between the epicondyle and the right arm’s epitrochlea. For this evaluation, participants were standing with the elbow bent at 90°. The distance between the femoral condyles (medial and distal) was considered the femur’s diameter, which evaluation was performed in a sitting position with the right knee bent at 90° [43, 44].

#### Skinfold thickness

Four skinfolds were considered to determine the participants’ somatotype: tricipital, subscapular, supra-iliac, and mid-calf [43–45].

#### Body Mass Index (BMI)

The BMI’s interpretation was made according to anthropometric standards to evaluate nutritional status [46].

#### Percentage of fat (%)

The fat percentage was evaluated through impedance measurement with the Tanita Inner Scan BC-554® digital scale.

#### Waist-Hip Index (WHI)

The WHI was obtained by dividing the waist perimeter, measured at a point equidistant from the lower edge of the last rib and the iliac crest, by the perimeter of the hips, measured at the greatest prominence of the buttocks [44, 47].

### 12 weeks without mandatory physical training

In regular class periods, the naval cadets had an average of two hours of daily mandatory physical training (Monday through Saturday). This physical training was mandatory and considered loads with the orientation to all physical capacities (strength, power, flexibility, speed, aerobic capacity and aerobic power). However, upon leaving school, whether for vacation or unplanned situations such as the current COVID 19 pandemic [30], the physical training regimen was not mandatory. During the 12 weeks without mandatory physical training, the naval cadets voluntarily took part in walking, cycling, and ball games, among other activities.

### Standardized warm-up

For both the first and the second evaluation of the 12MRT, the warm-up consisted of 10 minutes of jogging, then 5 minutes of ballistic movements of the lower limb (adduction, abduction, flexion, and extension of hips, and flexion and extension of knees and ankles). To finish, participants performed three 80-meter accelerations. After this warm-up and before running the 12MRT, there was a 5-minute break.

### 12-minute race test

The evaluation of the 12MRT was carried out on a 400-meter athletic track. Before the evaluation, participants were instructed to perform as much distance as possible within the test’s 12 minutes. During the application of the test, the participants received verbal incentives from the researchers. The distance achieved in meters was converted into kilometers, and then the VO_2_ max was obtained through the following equation [48]:

$$VO_{2}\;max\;(mLO_{2}\cdot kg^{–1}\cdot min^{–1})=(22.351\;x\;distance\;in\;kilometers)–11.288$$

### Statistical analysis

Descriptive data are presented as means and standard deviations. The normal distribution of the data was confirmed by the Shapiro-Wilk test (p > 0.05). A t-test for related samples was used to evidence changes between test and post-test, while Cohen’s d-test was used to determine the effect size (ES): < 0.2: negligible; 0.2–0.6: small; 0.6–1.2: moderate; 1.2–2.0: large; and > 2.0: very large. The association between the VO_2_ max and the fat percentage of the participants was quantified through the Pearson’s correlation coefficient (r) and according to the following scale: 0.00–0.09: trivial; 0.10–0.29: small; 0.30–0.49: moderate; 0.50–0.69: large; 0.70–0.89: very large; 0.90–0.99; almost perfect; and 1.00: perfect [49]. The statistical analysis was performed with SPSS software version 22.0 (SPSS, Chicago, IL, USA). After adjusting the numerous comparisons for supervising the false-positive risk results (0.05 / 18 = 0.002) [50], the alpha level for all statistical analyses was p < 0.002.

## Results

After applying the t-test for the physical performance variables, both the distance in the 12MRT and the VO_2_ max showed significant decreases at the end of the 12-week mandatory non-physical training period (p < 0.001, ES = 0.34). The values and changes observed in the VO_2_ max and anthropometric parameters are presented in Table 2.

**Table 2:** Mean values and SD before and after 12 weeks without mandatory physical training (n = 38).

	Test mean ± SD	Post test mean ± SD	Related differences							
			Mean	SD	SEM	95% confidence interval		t	p	d
						Lower	Upper			
**Weight (kg)**	67.1 ± 8.0	67.5 ± 8.3	-0.32	1.78	0.28	-0.91	0.25	-1.13	ns	0.01
**BMI (kg/m**^**2**^**)**	22.5 ± 1.7	22.7 ± 1.8	-0.16	0.58	0.09	-0.35	0.02	-1.78	ns	0.10
**% Fat**	14.9 ± 5.2	14.9 ± 5.4	0.05	1.24	0.2	-0.35	0.46	0.26	ns	0.01
**WHI**	0.84 ± 0.05	0.83 ± 0.04	0.00	0.03	0.00	0.00	0.01	0.71	ns	0.08
**WHeI**	0.46 ± 0.03	0.46 ± 0.02	0.00	0.01	0.00	0.00	0.00	0.84	ns	0.08
**Tricipital skinfold (mm)**	11.1 ± 3.9	11.8 ± 4.0	-0.69	1.83	0.29	-1.29	-0.09	-2.34	**ns**	0.18
**Subscapular skinfold (mm)**	10.7 ± 3.1	10.9 ± 3.0	-0.26	1.32	0.21	-0.7	0.17	-1.22	ns	0.09
**Suprailiac skinfold (mm)**	9.4 ± 3.4	10.4 ± 3.8	-0.97	3.02	0.49	-1.97	0.01	-1.99	ns	0.27
**Mid-calf skinfold (mm)**	10.2 ± 4.6	9.9 ± 3.6	0.30	2.34	0.37	-0.46	1.07	0.79	ns	0.07
**Arm circumference (cm)**	31.6 ± 2.9	31.8 ± 3.0	-0.16	1.62	0.26	-0.7	0.36	-0.62	ns	0.06
**Leg circumference (cm)**	36.7 ± 2.0	36.8 ± 2.1	-0.11	0.77	0.12	-0.37	0.13	-0.91	ns	0.06
**Humerus diameter**	6.77 ± 0.42	6.76 ± 0.40	0.00	0.15	0.02	-0.04	0.05	0.21	ns	0.01
**Femur diameter**	9.76 ± 0.53	9.69 ± 0.52	0.06	0.17	0.02	0.01	0.12	2.4	ns	0.13
**Endomorphic component**	3.12 ± 0.96	3.32 ± 1.00	-0.20	0.55	0.08	-0.38	-0.02	-2.32	**ns**	0.21
**Mesomorphic component**	5.07 ± 0.96	5.10 ± 0.93	-0.02	0.39	0.06	-0.15	0.10	-0.41	ns	0.03
**Ectomorphic component**	2.51 ± 0.76	2.44 ± 0.77	0.06	0.27	0.04	-0.02	0.15	1.39	ns	0.08
**12MRT (m)**	3100.8 ± 348.6	2978.1 ± 364.7	122	115	18.6	84.9	160.5	6.57	***	0.34
**VO**_**2**_ **max (mLO**_**2**_**·kg**^**–1**^**·min**^**–1**^**)**	56.6 ± 6.9	54.2 ± 7.2	2.45	2.3	0.37	1.69	3.21	6.57	***	0.34

SD: standard deviation; SEM: standard error of the mean; WHI: waist-hip index; WHeI: waist-height index; BMI: muscle mass index; kg/m^2^: kilograms per square meters; 12MRT: 12-minute race test; mm: millimeters; cm: centimeters; m: meters; VO_2_ max: maximum oxygen consumption; mLO_2_·kg^–1^·min^–1^: milliliters of oxygen per kilogram of body mass per minute

*** p < 0.002; ns: not significant; d: Cohen’s d.

**Fig 2:**
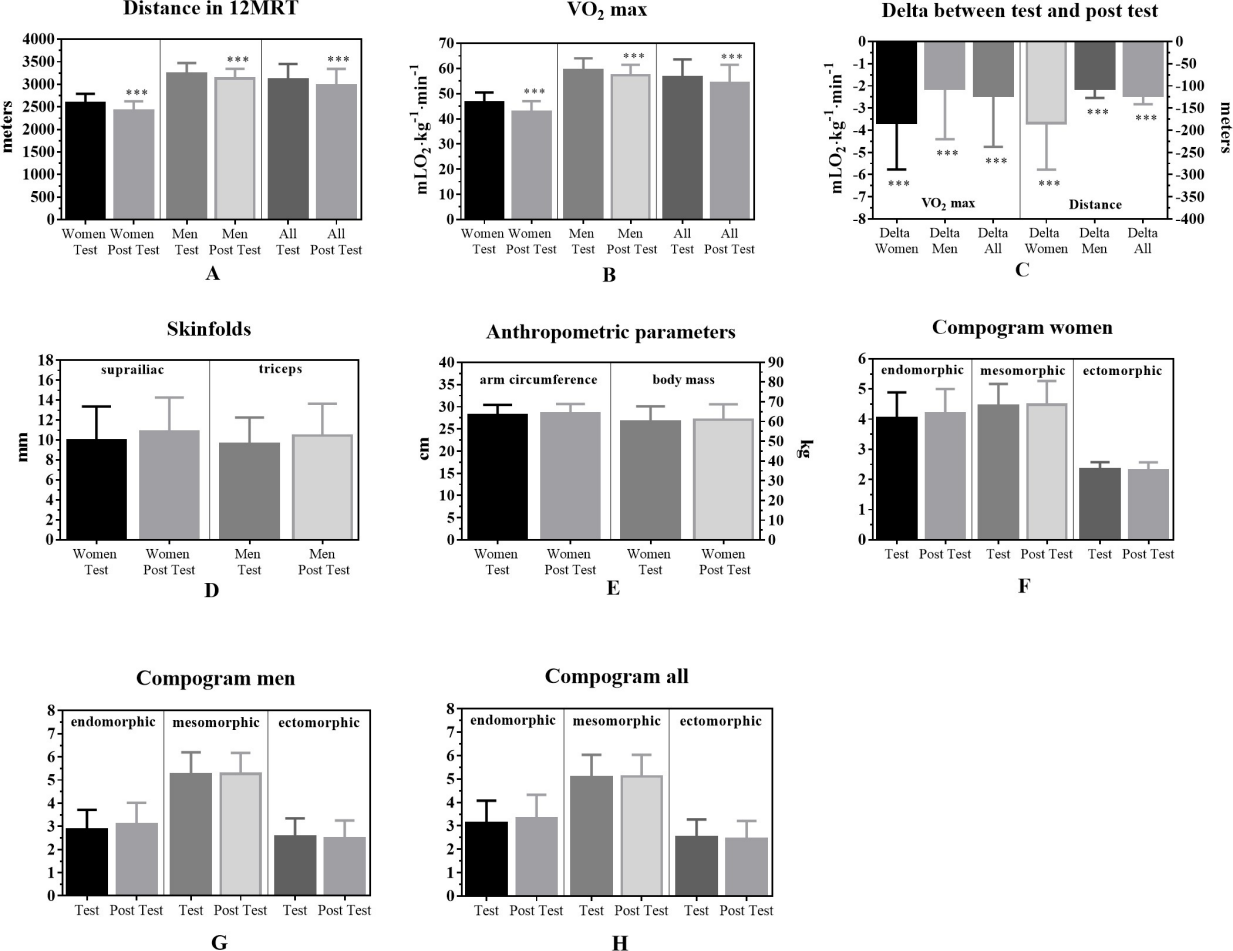
Changes in VO_2_ max and anthropometric parameters before and after 12 weeks without mandatory physical training. 12MRT: 12-minute race test; mLO_2_·Kg^–1^·min^–1^: milliliters of oxygen per kilogram of body mass per minute; mm: millimeters; cm: centimeters; kg: kilograms; ***: p < 0.002.

When analyzing by sex, distance in 12MRT and VO_2_ max showed significant decreases after 12 weeks without mandatory physical training in both men (p < 0.001, ES = 0.47) and women (p < 0.001, ES = 0.90). Likewise, both the tricipital skinfold thickness in men (test: 9.64 ± 2.62; post test 10.46 ± 3.17; t = -2.39; p = 0.02; ES = 0.28) and the supra-iliac fold in women (test: 9.95 ± 3.41; post test 10.85 ± 3.41; t = -3.21; p = 0.015; ES = 0.26) showed increases after 12 weeks without mandatory physical training. Finally, women increased arm circumference (test: 28.08 ± 2.34; post test 28.51 ± 2.09; t = -2.37; p = 0.04; ES = 0.19) and body weight (test: 59.86 ± 7.84; post test 60.83 ± 7.93; t = -2.86; p = 0.02; ES = 0.12) in this period of time. The values and changes observed in these variables are presented in Fig 2.

**Fig 3:**
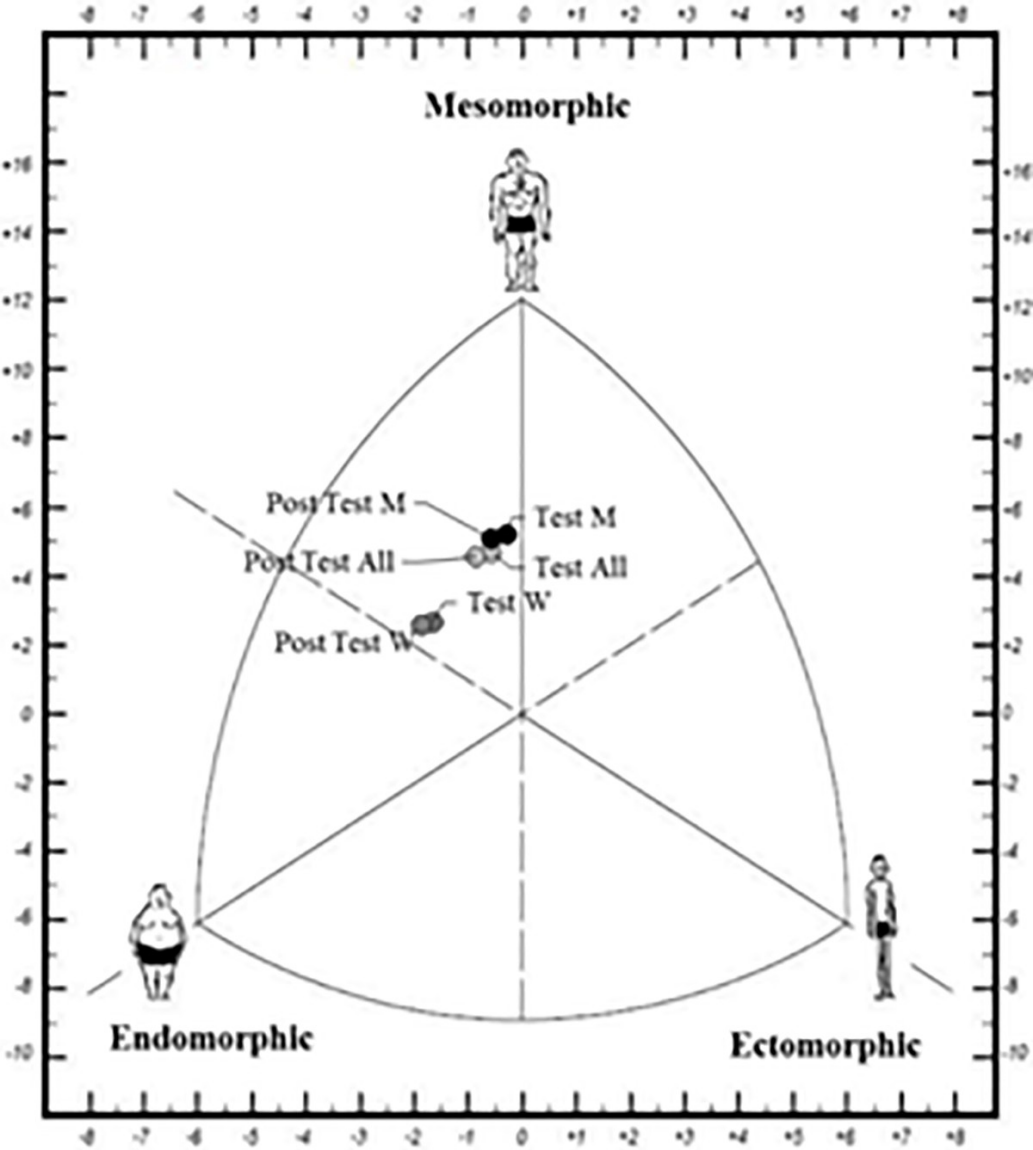
Somatotype before and after 12 weeks without mandatory physical training.

At the end of the period without mandatory physical training, an increase in the endomorphic component was observed (p = 0.02, ES = 0.21). The graphical representation of the somatotype, before and after 12 weeks without mandatory physical training, for all participants, men, and women, is presented in Fig 3.

**Fig 4:**
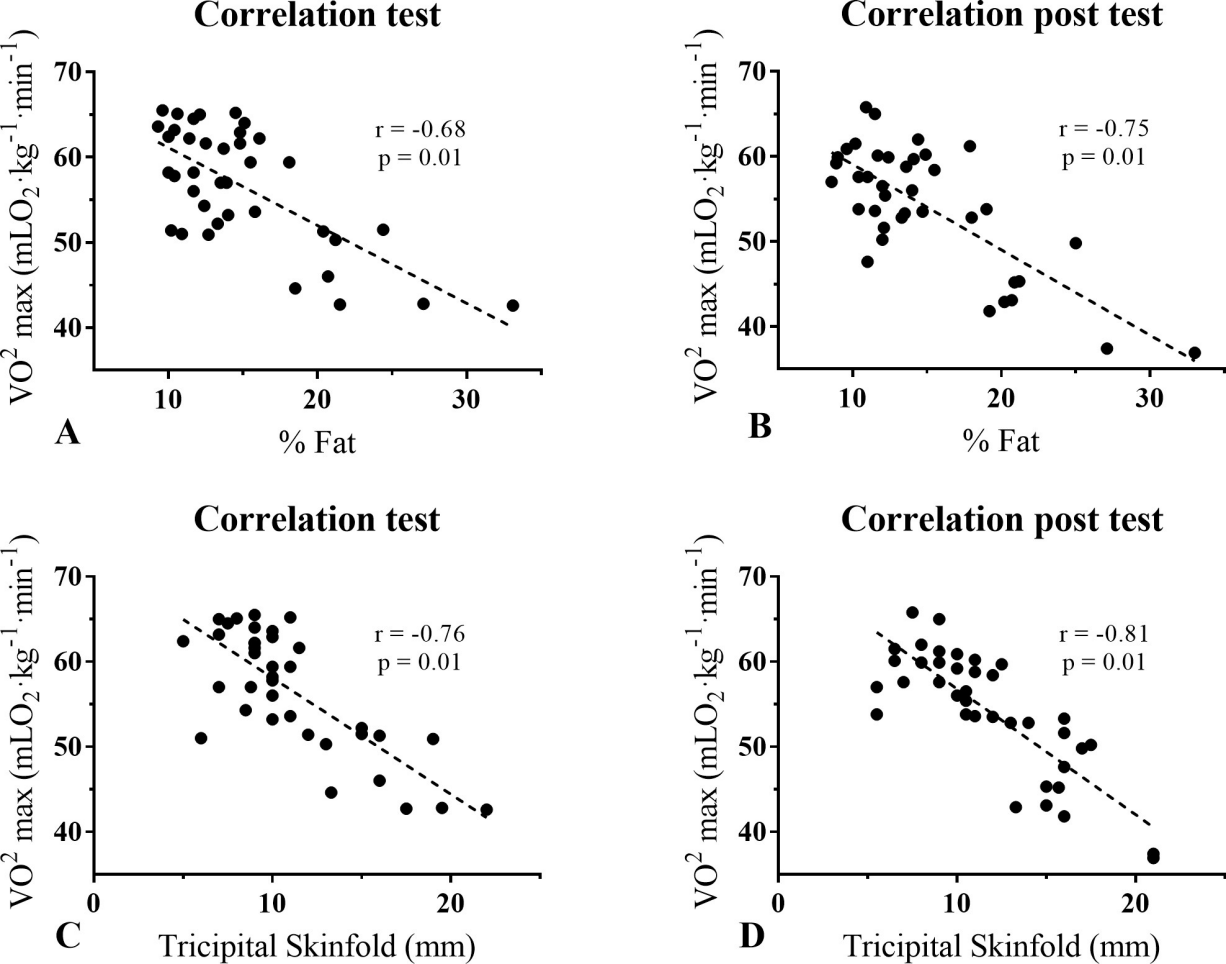
Correlation between VO_2_ max and anthropometric parameters before and after 12 weeks without mandatory physical training. mLO_2_·Kg^–1^·min^–1^: milliliters of oxygen per kilogram of body mass per minute; % fat: percentage of fat; mm: millimeter.

By relating the VO_2_ max to the fat percentage participants, a large, negative correlation was observed between both variables (r = -0.69, p = 0.01). At the end of the 12 weeks without mandatory physical training (post-test), a very large, negative correlation was observed between VO_2_ max and the participants’ fat percentage (r = -0.75, p = 0.01). By relating the VO_2_ max to the tricipital skinfold participants, a very large, negative correlation was observed between both variables (r = -0.76, p = 0.01). At the end of the 12 weeks without mandatory physical training (post-test), a very large, negative correlation was observed between VO_2_ max and the participants’ tricipital skinfold (r = -0.81, p = 0.01). The graphic representation of these analyses is presented in Fig 4.

## Discussion

Concerning this study’s primary objective, the variables of VO_2_ max and anthropometric parameters showed changes after the 12 weeks without mandatory physical training in naval cadets from 18 to 25 years old. The findings revealed that the analysis initial point relates physical training to quality of life [6, 8] and sports performance [1–3]. In this way, detrimental physiological changes and a decline in performance observed after a period without physical training can be reversed by applying correct training loads and professional supervision [17]. Specifically, the present study’s findings showed a significant decrease in the VO_2_ max of naval cadets, both men and women, after 12 weeks without mandatory physical training (p < 0.001, ES = 0.34). Similarly, Liguori et al. [37] determined changes in VO_2_ max after a vacation period without mandatory training; at the end of the vacation period, the researchers reported significant decreases in relative (p = 0.009) and absolute (p = 0.001) VO_2_ max in both men and women. Likewise, Sotiropoulos et al. [33] evaluated changes in VO_2_ max after a four-week transition period in soccer players. The experimental group (EG) conducted a directed training program, while the control group (CG) executed a free training program. At the end of the research, the EG decreased from 57.66 ± 2.56 to 56.85 ± 2.52 mLO_2_·kg^-1^·min^-1^. In contrast, the CG decreased from 58.08 ± 2.60 to 54.52 ± 2.80 mLO_2_·kg^-1^·min^-1^. Additionally, the researchers reported significant decreases in VO_2_ max when comparing the EG to the CG in the post-test (t = 16.06; p < 0.0001). Likewise, the endomorphic somatotype has a greater fat mass than the mesomorphic and ectomorphic somatotype [43], and subjects with endomorphic predominance have shown a lower VO_2_ max than subjects with a mesomorphic or ectomorphic predominance (endomorphic: 37.3 ± 0.77; mesomorphic: 40.2 ± 0.46; and ectomorphic: 43.5 ± 0.52) [51]. For this reason, the increase in the endomorphic component observed in naval cadets after 12 weeks without mandatory physical training could condition the decrease of VO_2_ max at the end of this period (p < 0.001, TE = 0.34). However, it is important to analyze the ES for each variable studied, which allows us to observe each phenomenon’s degree of presence, independent of the alpha level calculated [52]. In this study, like in research by Parpa & Michaelides [24], all ES in the tests with significant differences in VO_2_ max, including men and all data analysis, oscillated between 0.2–0.6. This was considered a small effect. On the other hand, the significant differences in women had an ES between 0.6–1.2 (which was considered as a moderate effect). Furthermore, the large and negative correlation between VO_2_ max and the fat percentage observed in the test (r = -0.69, p = 0.01) increased after the period without mandatory physical training (r = -0.75, p = 0.01). Up to this point, the decrease in VO_2_ max has been attributed to two leading causes; on the one hand, a transition period without mandatory and controlled physical training, while on the other hand, an increase in fat mass, reflected in the endomorphic component of naval cadets [51].

Periods without physical training have also been associated with a decrease in muscle cross-section [34]. This unfavorable consequence could be related to lower levels of muscle strength [35]. In this case, Koundourakis et al. [31] examined the effects of six weeks without physical training on performance parameters in soccer players; at the end of the study, the researchers reported significant decreases in both squat jump (Team A: 39.70 ± 3.32 vs 37.30 ± 3.08 kg; p < 0.001; Team B: 41.04 ± 3.34 vs 38.18 ± 3.03 kg; p < 0.001) and countermovement jump (Team A: 41.04 ± 3.99 vs 39.13 ± 3.26%; p < 0.001); Team B: 42.82 ± 3.60 vs 40.09 ± 2.79 kg; p < 0.001) in both experimental groups. The researchers also concluded that the observed reductions in jumping ability (considered to be a negative effect) could be related to mismatches of rapidly contracting muscle fibers [25, 53]. In parallel, the endomorphic somatotype has lesser muscle mass than the mesomorphic and ectomorphic somatotype [43]. In turn, Miroshnichenko et al. [51] showed a high correlation between the predominance of the mesomorphic component and VO_2_ max. Likewise, an increase in the endomorphic component and lower muscle mass could be associated with a lower VO_2_ max of the participants. Therefore, an increment of the endomorphic component in naval cadets may decrease the lower extremities’ strength, generating biomechanical and neuronal changes [54]. These last changes could affect the economy of the race [55] and, consequently, decrease the performance in 12MRT (p < 0.001, ES = 0.34). Although the evidence shows the negative influence of periods without training on strength and muscular power [31, 35], mainly due to loss of muscle mass [34, 51], the present study did not consider assessing naval cadets’ anaerobic capacity. Therefore, the possible effects of 12 weeks without mandatory physical training on strength or power in both the lower and upper extremities should be considered in future studies.

On the other hand, this study also showed increases in some anthropometric parameters after 12 weeks without mandatory physical training, specifically in the tricipital skinfold thickness in men (p = 0.02, ES = 0.18), arm circumference in women (p = 0.04, ES = 0.19) and the endomorphic component in both men and women (p = 0.02, ES = 0.25). In this sense, evidence shows that a period without physical training leads to increased fat mass and a decreased lean mass [31–33]. Also, the tricipital fold, together with the subscapular and suprailiac folds, are anthropometric indicators with a high explanatory power of VO_2_ max in both sexes [56]. We evidenced that those naval cadets with a higher tricipital fold had a reduced VO_2_ max (Test: r = 0.76, p = 0.01; post test: r = 0.81, p = 0.01). Likewise, an elevated tricipital fold conditions an elevated endomorphic component [42]. Consequently, anthropometric parameters influence cardiorespiratory fitness, independent of sex, age, and obesity level [57]. Related to this, Sotiropoulos et al. [33] evaluated changes in body weight and body fat percentage after a four-week transition period in soccer players (The EG conducted a directed training program and the CG a free training program). At the end of the study, the EG increased from 78.14 ± 4.77 to 78.74 ± 5.00 kg, while the CG increased from 76.48 ± 2.65 to 77.90 ± 2.82 kg (t = -4.91; p < 0.005); and, also reported increased percentage of body fat (EG from 7.92 ± 1.68 to 8.17 ± 1.81%; CG from 7.77 ± 1.79 to 8.59 ± 1.80%; t = -8.42; p < 0.005). On the other hand, Ormsbee et al. [58] examined the effect of five weeks without physical training on body composition in swimmers. At the end of the study, significant differences were observed in body weight (68.96 ± 9.7 vs. 69.8 ± 9.8 kg; p = 0.03), fat mass (14.7 ± 7.6 vs. 16.5 ± 7.4 kg; p = 0.001), and waist circumference (72.7 ± 3.1 vs. 73.8 ± 3.6 cm; p = 0.03). Also, Koundourakis et al. [31] examined the effects of six weeks without physical training on the body composition of soccer players; at the end of the study, the researchers reported significant increases in both body weight (Team A: 77.60 ± 5.88 vs. 79.13 ± 6.16 kg; p < 0.001; Team B: 77.89 ± 8.75 vs. 79.49 ± 8.95 kg; p < 0.001) and in the fat percentage (Team A: 9.2 ± 3.33 vs. 11.01 ± 4.11%; p < 0.001; Team B: 9.43 ± 3.55 vs. 10.40 ± 4.08 kg; p < 0.001) in both experimental groups.

Although some studies have established the body composition of armed forces personnel in some countries [59] and anthropometric changes have been documented concerning soldiers’ physical training [60], the effects of 12 weeks without mandatory physical training on anthropometric parameters have not been reported for naval cadets. Consequently, in connection with the studies referred to above, our study’s findings show the importance of verifying and controlling body composition after a period without mandatory physical training in naval cadets [61], especially somatotype indicators [43]. However, it is essential to mention that the present study did not control the participants’ caloric intake [62]. For this reason, we are not sure that the changes in anthropometric parameters were only due to a decrease in physical training [63–65]; there is a possibility that higher caloric intake, above the daily energy needs, has also influenced these physical changes [62, 66].

Finally, the data show that VO_2_ max is an essential parameter of the physical condition [38], and a higher VO_2_ max allows the efficient performance of physical tasks associated with military personnel [13, 60]. It has also been demonstrated that subjects with a higher percentage of body fat have lower VO_2_ max, lower strength levels, and lower fatigue tolerance [67]. As demonstrated in this study, a vacation period without mandatory physical training generates decreases in the VO_2_ max [37] and negatively affects anthropometric parameters [51]. Therefore, the vacation periods must be adapted into a transition phase [24, 25]. In this way, with controlled and directed physical training, both athletes and naval cadets will have optimal physical recovery and maintenance; this condition will allow them to face better the next cycle of physical training [23].

One of the limitations of this study was the sample used. As mentioned above, the sample was by convenience, which would not allow us to generalize the data. However, armed forces personnel are more homogeneous in body structure [68] and eating behavior [69]. For this reason, in this specific case, the results could be generalized to this population.

## Conclusions

Twelve weeks without mandatory physical training significantly decreases the VO_2_ max in naval cadets from 18 to 25 years old. Simultaneously, the same period without mandatory training increases skinfold thickness and the endomorphic component in this population.

## Practical applications

After evidence of decreases in VO_2_ max and negative increases in some anthropometric parameters after 12 weeks without mandatory physical training, it is suggested that training loads in the transition phase [25], whether due to vacations [28] or to unforeseen events [30].
